# Prosthetic knee joint infection caused by *Aspergillus penicillioides*: a case report

**DOI:** 10.1186/s12879-025-12091-y

**Published:** 2025-11-24

**Authors:** Haruki Nakano, Kenjiro Fujimura, Tomohide Okinaka, Takashi Yaguchi, Yusuke Kubo, Takashi Matono, Toshihiko Hara

**Affiliations:** 1 Department of Orthopedic Surgery, Aso Iizuka Hospital, 3-83, Yoshio, Iizuka, Fukuoka Prefecture Japan; 2 Department of Infectious Diseases, Aso Iizuka Hospital, Iizuka, Fukuoka Japan; 3 Medical Mycology Research Center, Chiba University, Chiba, Japan

**Keywords:** *Aspergillus penicillioides*, Periprosthetic joint infection (PJI), Fungal infection

## Abstract

**Background:**

Periprosthetic joint infection (PJI) is a challenging complication of joint arthroplasty, and fungal pathogens account for only 1% of all such cases. Among these, *Aspergillus*-related PJI is exceedingly rare, and to date, no standardized treatment guidelines have been established.

**Case presentation:**

An 86-year-old woman presented with right knee pain 14 years after total knee arthroplasty. PJI was suspected based on the initial characteristics of the joint fluid, prompting surgical irrigation and debridement. Although the initial bacterial cultures before antibiotic administration were negative, fungal growth was observed in two intraoperative samples. Detailed analysis identified the causative organism as *Aspergillus penicillioides* after 8 weeks. The patient achieved stabilization of C-reactive protein levels with voriconazole treatment, without undergoing implant removal.

**Conclusions:**

We present a case of *Aspergillus penicillioides*-related PJI, which, to our knowledge, has not been previously reported in PJIs. This case highlights the diagnostic challenges posed by rare fungal PJIs, particularly those caused by *Aspergillus penicillioides*. It is important to maintain clinical vigilance for rare fungal PJIs, as certain species, such as *Aspergillus penicillioides*, are slow-growing and difficult to identify with standard culture techniques.

**Clinical trial number:**

Not applicable.

## Background

Periprosthetic joint infection (PJI) is one of the most challenging complications associated with joint arthroplasty and is sometimes difficult to diagnose. Fungal pathogens account for only 1% of all PJIs, with *Candida* species representing approximately 80–90% of these cases [1, 2]. Indeed, while much of the literature on fungal PJI is focused on *Candida*-related cases, *Aspergillus*-related PJI is exceedingly rare in all joints, including the knee, with only limited reports available, and no universally accepted treatment guidelines currently exist [3]. The rarity of *Aspergillus*-related PJIs and their atypical clinical presentation make timely diagnosis and appropriate treatment particularly complex. Understanding the characteristics, diagnostic challenges, and management strategies of these infections is essential for improving the outcomes in affected patients. We report a rare case of *Aspergillus*-induced periprosthetic knee joint infection with several literature reviews.

## Case presentation

**Fig. 1:**
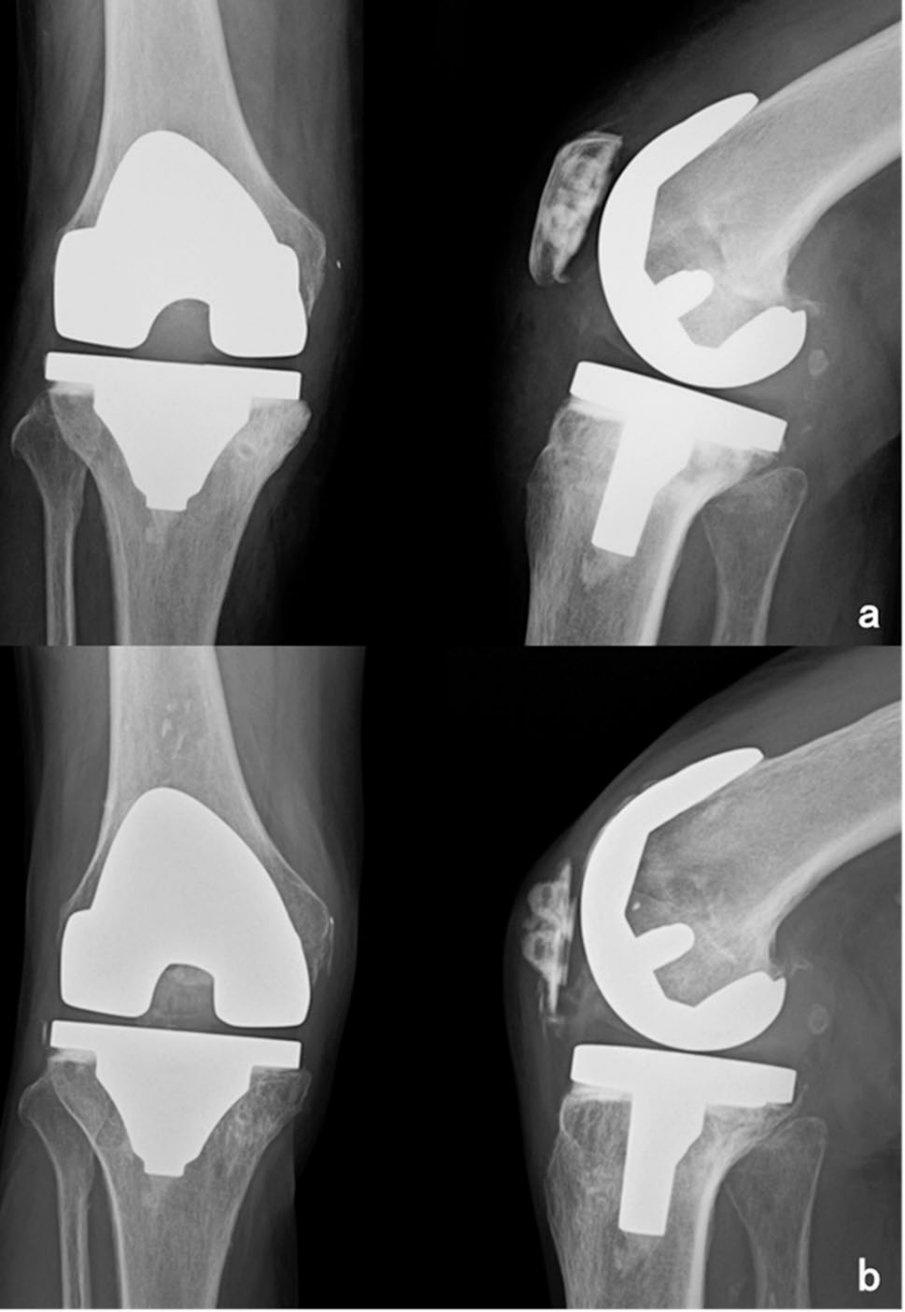
Radiographs of the right knee. (**a**) Radiograph obtained immediately after total knee arthroplasty, (TKA) using the Scorpio NRG PS implant, (Stryker, Marwah, NJ, USA), (**b**) Radiograph obtained after the patient presented with knee pain. Fourteen years post-surgically, no loosening was observed

**Fig. 2:**
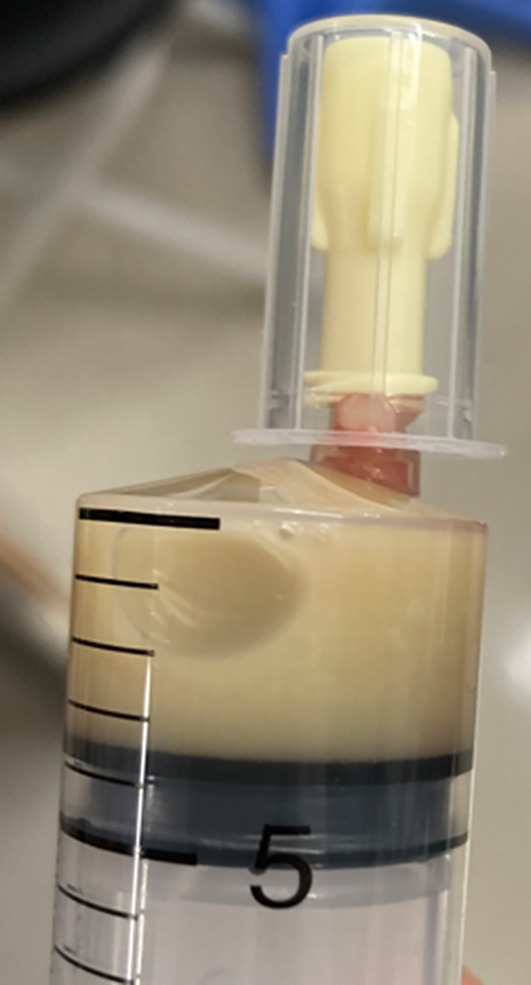
Photographic clinical view of the synovial fluid

An 86-year-old woman presented to our department with right knee pain that had begun the previous day. Fourteen years earlier, she underwent right total knee arthroplasty (TKA) at our institution. Her medical history included hypertension, chronic renal failure (creatinine: 1.67 mg/dL), dyslipidemia, a non-functional adrenal tumor, arteriosclerosis obliterans (with an ankle-brachial index of 0.85 on the left and undetectable on the right), and lumbar spinal canal stenosis. She had no history of autoimmune disease and was not taking any immunosuppressants such as corticosteroids. On examination, she presented with mild warmth, tenderness, and swelling around the right knee joint. Fever or redness overlying the joint was not observed. The results of initial haematological and biochemical investigations revealed a white blood cell (WBC) count of 6,890/µL and a C-reactive protein (CRP) level of 176.8 mg/L. Radiography revealed no signs of implant loosening (Fig. 1). A knee joint aspiration revealed a small amount of purulent and cloudy fluid (Fig. 2). The aspirated synovial fluid volume was limited, which precluded reliable quantitative analysis of the synovial WBC count in our laboratory. Therefore, only qualitative microscopy was performed, showing no crystals and a negative Gram stain. The synovial glucose level was measured using a point-of-care glucometer and was < 40 mg/dL, compared with a concurrent blood glucose level of 88 mg/dL. These findings supported the suspicion of PJI. Consequently, surgical irrigation and debridement were planned the following day. Both synovial fluid and blood samples were obtained for culture testing, after which preoperative administration of cefazolin sodium (2 g every 12 h) was promptly initiated.

**Fig. 3:**
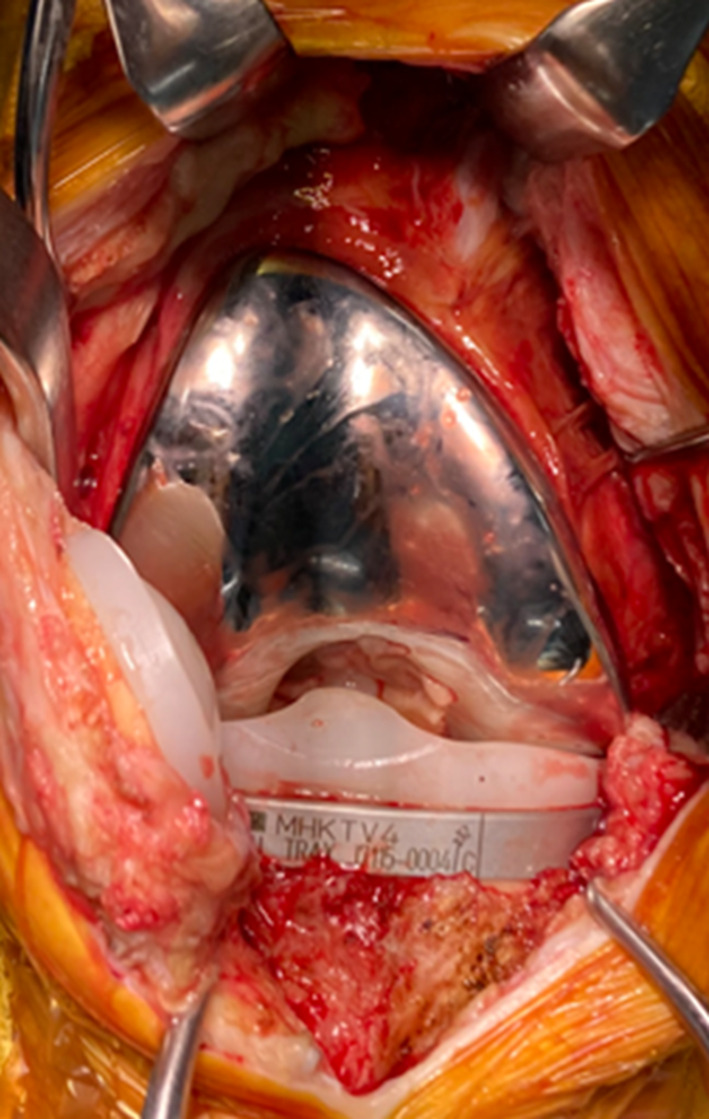
Intraoperative photograph

**Fig. 4:**
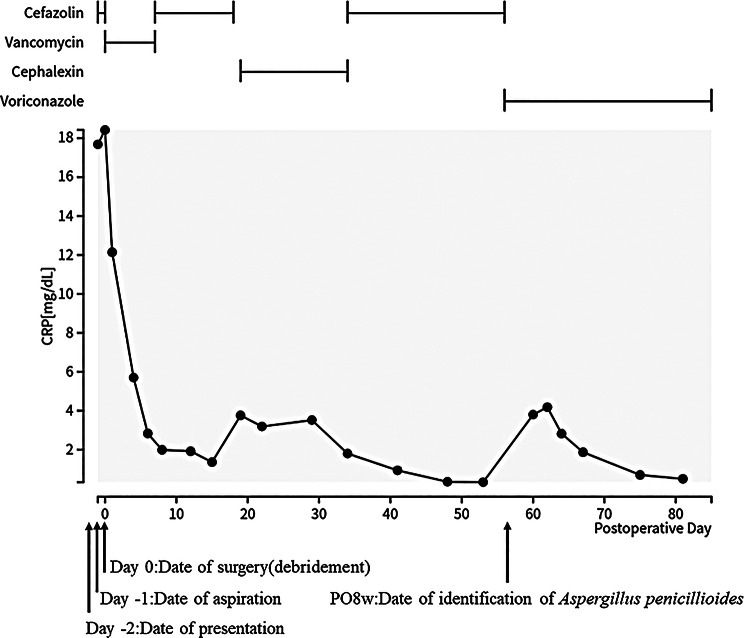
Duration of each antibiotic use and the corresponding C-Reactive Protein (CRP) values

**Fig. 5:**
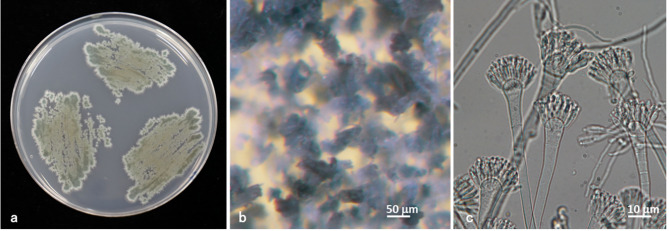
*Aspergillus penicillioides* IFM 68,718 colonies, Colonies with the isolate spread on potato-glucose agar (PDA) + 5%NaCl, 25 C, 7d (**a**) Conidial heads (**b**) Aspergilla (**c**)

The intraoperative findings revealed only a small amount of synovial fluid without overt purulence. No bone destruction, synovial hyperplasia, or implant loosening were observed (Fig. 3). Tissue samples were collected from three different sites and cultured. Thorough irrigation and debridement were performed and only the insert was exchanged. Despite the lack of typical signs of infection such as synovitis, postoperative treatment with vancomycin was initiated, considering the possibility of methicillin-resistant *Staphylococcus aureus* (MRSA) infection. One week post-surgery, no bacterial growth was observed in either the synovial fluid culture or blood culture. Despite de-escalation from vancomycin to cefazolin sodium (2 g every 12 h), the CRP levels continued to decrease steadily (Fig. 4). Two weeks after the surgery, fungal growth was observed in two independent intraoperative samples. Unfortunately, slow growth of the fungus made species identification a challenging task. Considering the potential side effects, antifungal therapy was not initiated prematurely given the unidentified causative organism. As the CRP levels steadily decreased, antibiotic treatment was switched from intravenous cefazolin sodium (2 g every 12 h) to oral cephalexin (500 mg three times daily after meals), considering the chance possibility of coinfection with typical bacteria. Five weeks postoperatively, although the fungal species had not yet been identified, CRP levels began to increase again, prompting a switch back to intravenous cefazolin sodium. Although CRP decreased after re-initiating intravenous cefazolin sodium, no cefazolin-susceptible bacterial pathogen was identified; therefore, a direct antibiotic effect cannot be confirmed, and spontaneous improvement or other confounding factors cannot be excluded. Six weeks postoperatively, cultures on Sabouraud medium showed extremely slow fungal growth. Grocott’s staining confirmed the presence of filamentous fungi. Further analyses were performed at the Medical Mycology Research Center at Chiba University. Eight weeks after surgery, the fungal pathogen was identified as *Aspergillus penicillioides* (Fig. 5). Based on its morphological characteristics, the isolate was presumed to be a xerophilic *Aspergillus* species. Therefore, genomic DNA was extracted from fungal biomass cultured on potato dextrose agar plates supplemented with 5% NaCl at 25 °C for 7 days using the PrepMan™ Ultra Sample Preparation Reagent (Thermo Fisher Scientific, Waltham, MA, USA).

The internal transcribed spacer (ITS) region, commonly used for fungal identification, and the calmodulin (CaM) gene, generally used for the identification of *Aspergillus* species, were amplified and directly sequenced from PCR products using the primer pairs ITS5/ITS4 (White et al., 1990) and cmd5/cmd6 (Hong et al., 2006), respectively [4, 5]. Sequencing reactions were performed with the BigDye Terminator v3.1 Cycle Sequencing Kit (Applied Biosystems, Foster City, CA, USA) on an ABI PRISM^®^ 3130 Genetic Analyzer (Applied Biosystems). A BLAST search (https://www.ncbi.nlm.nih.gov/blast/) of the obtained sequences revealed the following results. The ITS region sequence of this strain showed high similarity to *Aspergillus penicillioides* and *Aspergillus clavatophorus*. In contrast, the calmodulin gene sequence, which exhibits greater interspecific variation, showed 100% identity with multiple *Aspergillus penicillioides* strains deposited in the database and 99.8% identity with the type strain *Aspergillus penicillioides* NRRL 4548 (GenBank accession no. EF652024). It has been preserved as IFM 68,718 at the Medical Mycology Research Center, Chiba University, as part of the National Bio Resource Project in Japan. Although antifungal susceptibility could not be determined, antifungal therapy with voriconazole was initiated and intravenous administration of cefazolin sodium was discontinued. Because this species is xerophilic, the MIC could not be determined using the standard CLSI broth dilution method, which uses RPMI 1640 broth medium unsuitable for its growth. According to the current IDSA clinical practice guidelines for invasive aspergillosis, voriconazole is recommended as the first-line antifungal agent for infections caused by *Aspergillus* species, including osteomyelitis and septic arthritis [6]. Therefore, our choice of voriconazole was justified based on established recommendations, although antifungal susceptibility testing was not performed. The optimal duration of therapy remains undefined in the guidelines and was determined in accordance with the patient’s clinical course and general condition. Although mild pain persisted, the CRP level nearly normalized. The patient was transferred to another facility 12 weeks after surgery. Eight months postoperatively, the CRP level was 1.8 mg/L, and the clinical course was favorable. Unfortunately, the patient died of an unknown cause 11 months postoperatively, before voriconazole administration could be discontinued. According to her niece, the patient had been hospitalized for a respiratory illness unrelated to fungal infection or antifungal therapy prior to her death.

## Discussion and conclusions

Reports of *Aspergillus*-related PJI remain exceedingly rare. In a review by Karczewski et al. [7], 10 cases of *Aspergillus*-related PJI were summarized: four cases of *Aspergillus fumigatus*, three cases of *Aspergillus terreus*, two cases of *Aspergillus niger*, and one case of an unidentified *Aspergillus* species. The infection sites included four hips, four knees, one elbow, and one proximal interphalangeal arthroplasty site [7]. Kuthan et al. reported a case of left knee PJI caused by *Aspergillus clavatus* [8]. Diagnosis based solely on joint aspiration fluid remains particularly challenging, with only 2 of 11 cases identified through aspiration fluid alone. Most cases are diagnosed using surgical specimens [7, 8]. The present case was incorporated into the table with reference to the systemic summary of *Aspergillus*-related PJI cases reported by Karczewski et al. (2021) (Table 1) [7–17].

**Table 1:**
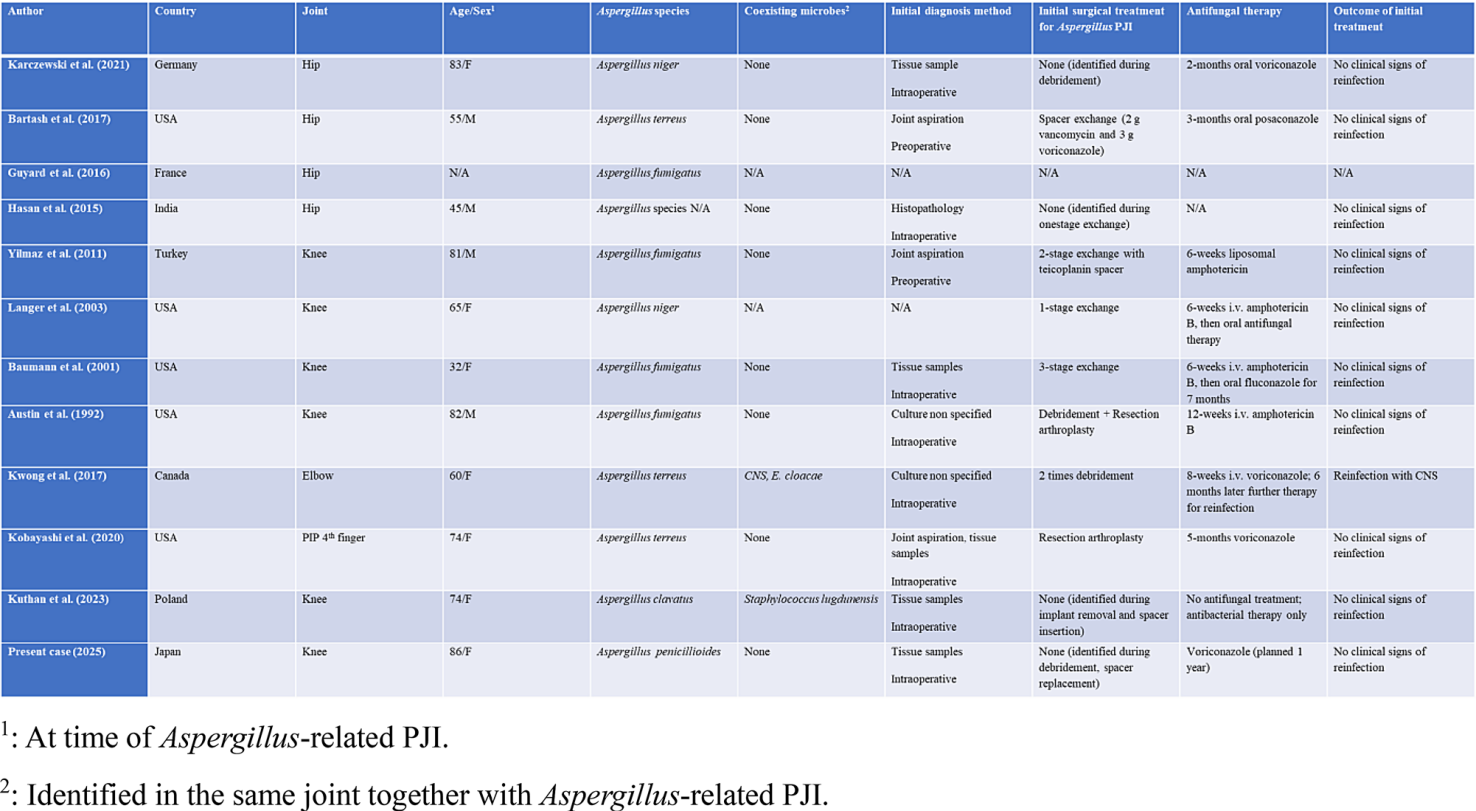
Summary of reported Aspergillus-related PJI and the present case. The cases from Karczewski through Kobayashi were summarized based on the data presented in the 2021 publication by Karczewski et al

*Aspergillus penicillioides*, the causative organism of PJI in this patient, was first described by Spegazzini in 1896 [18]. It is widely distributed in nature and is found in grains, dried fruits, baked goods, salted fish, spices, binocular lenses, and human skin [19]. This fungal species is characterized by poor and slow growth on standard fungal media, making its isolation and identification particularly challenging. Machowicz-Matejko et al. reported that the first colonies of *Aspergillus penicillioides* appeared on potato-glucose agar (PDA) media after 15–17 days [20]. The first report of disseminated aspergillosis caused by *Aspergillus penicillioides* in humans was published by Gupta et al. (2015) during the autopsy of an infant with cystic fibrosis [21]. Machowicz-Matejko et al. successfully isolated *Aspergillus penicillioides* from the human cornea [20]; however, to the best of our knowledge, no previous reports of *Aspergillus penicillioides*-related PJI have been reported.

According to the European Bone and Joint Infection Society (EBJIS) criteria, the isolation of a rare or highly virulent microorganism from a single sample warrants suspicion of PJI [22]. In our case, *Aspergillus penicillioides* was identified in two separate samples, strongly supporting its role as the causative pathogen. Although fungal PJIs are typically considered opportunistic infections, this case did not fit this paradigm. Tiwari et al. described a case of *Candida* PJI in a healthy young woman, highlighting that fungal PJI can occur in immunocompetent patients without any obvious risk factors [23].

Mixed bacterial and fungal infections have often been reported, with bacterial biofilm formation potentially protecting the fungal pathogens from antimicrobial therapy [24]. In the present case, no bacterial pathogens were identified; however, ruling out mixed infections was challenging. Administration of antibiotics before surgery may have masked cefazolin-sensitive bacteria, as culture-negative PJIs are common. The decrease in CRP levels with antibiotic therapy alone suggests the possibility of a mixed infection. However, the absence of typical signs of synovitis and the lack of increased accumulation of cloudy joint fluid suggest that it may have been solely a fungal infection. Furthermore, in cases like this one, where the clinical course and intraoperative findings are atypical, it would be reasonable to suspect the involvement of atypical microorganisms, including fungi, anaerobic bacteria, and non-tuberculous acid-fast bacteria.

Two-stage revision surgery is widely regarded as the standard treatment for PJI [25]. Klatte et al. reported a reinfection rate of 10% in 10 cases, and single-stage revision following fungal periprosthetic infection was feasible with an acceptable rate of satisfactory outcomes [26]. Ji et al. reported that three reinfections among 11 patients managed with one-stage revision and single-stage revision can be fairly effective for achieving acceptable functional outcomes [27]. In our case, PJI was initially suspected based on the preoperative synovial fluid characteristics; surgical irrigation and debridement were performed accordingly. However, intraoperative findings revealed that the intra-articular condition was cleaner than anticipated, with no convincing evidence of implant loosening. Given the possibility of an atypical or fungal infection, postoperative antibiotic therapy was continued. The clinical course remained stable without any recurrence of symptoms, and *Aspergillus penicillioides* was identified as the causative organism eight weeks postoperatively. Despite this finding, a two-stage revision was not planned, as there were no clinical signs such as local heat, redness, or swelling. Furthermore, considering the patient’s advanced age and high surgical risk, antifungal therapy was continued with the policy that implant removal would be reconsidered only if clear evidence of infection recurrence emerged. The patient was followed up for 8 months postoperatively, during which the CRP level remained at 1.8 mg/L, and the pain could be managed well without necessitating implant removal. Unfortunately, the patient died before voriconazole treatment could be discontinued, preventing the observation of long-term post-treatment outcomes. We acknowledge that preservation of the prosthesis in this case may have been partly due to chance. This outcome appears to have been influenced by several factors, including prompt and thorough irrigation and debridement performed after the onset of symptoms, the effectiveness of the administered antibacterial and antifungal therapies, the absence of any immunosuppressive conditions in the patient aside from advanced age, and the fact that her level of daily activity was relatively low.

Two possible routes of infection can be considered in this case: (1) perioperative contamination during the initial arthroplasty, and (2) hematogenous seeding from an unidentified source. Although the exact infection route remains speculative, perioperative contamination appears to be the most plausible hypothesis for a late-onset PJI. *Aspergillus penicillioides* is a xerophilic fungus widely distributed in the environment, including grains, dried foods, house dust, and even indoor walls. Because of its remarkable tolerance to dry conditions, this organism can survive for prolonged periods in the environment. While modern operating rooms maintain a high level of sterility, it is virtually impossible to completely eliminate the risk of airborne fungal spores settling in the surgical field. Therefore, it is conceivable that contamination during the perioperative period might have contributed to infection development years after the initial surgery.

This case report has several limitations. First, antifungal susceptibility testing was not performed, partly because *Aspergillus penicillioides* possesses xerophilic characteristics, making culture-based testing technically difficult. Second, long-term follow-up was not possible because the patient passed away 11 months postoperatively. Finally, we were not in an environment where advanced diagnostic tests could be easily performed; therefore, in this case, we could not completely rule out the possibility of coinfection. However, we believe that no bacterial coinfection was present. Although the evidence is admittedly limited, this assumption is supported by the fact that antibacterial therapy was promptly discontinued once *Aspergillus penicillioides* was identified, and the patient’s CRP levels subsequently decreased to nearly normal values with antifungal monotherapy alone and remained stable thereafter. Despite these limitations, this case contributes to the limited literature on fungal PJI, especially those caused by *Aspergillus penicillioides*.

In conclusion, this is the first report of *Aspergillus penicillioides*-related PJI. *Aspergillus*-related PJI are exceedingly rare, and their diagnosis and treatment remain unstandardized. When PJI is suspected and conventional cultures fail to identify the pathogen, clinicians should consider the possibility of fungal infections involving organisms that grow only under specialized conditions. As in this case, it is noteworthy that fungi and acid-fast bacteria, which require specific culture conditions, can be among the causative microorganisms in patients with culture-negative PJI. Collaboration with infectious disease specialists and use of appropriate microbiological techniques, including PCR methods, may be warranted for improving patient outcomes.

## Data Availability

The datasets used and/or analyzed in the current study are available from the corresponding author upon reasonable request.

## Abbreviations

BLAST: Basic local alignment search tool
CRP: C-reactive protein
EBJIS: the European Bone and Joint Infection Society
MRSA: Methicillin-resistant Staphylococcus aureus
PDA: Potato-glucose agar
PJI: Periprosthetic joint infection
TKA: Total knee arthroplasty
WBC: White blood cell
